# Chronic Exposure to Arsenic and Markers of Cardiometabolic Risk: A Cross-Sectional Study in Chihuahua, Mexico

**DOI:** 10.1289/ehp.1408742

**Published:** 2015-06-12

**Authors:** Michelle A. Mendez, Carmen González-Horta, Blanca Sánchez-Ramírez, Lourdes Ballinas-Casarrubias, Roberto Hernández Cerón, Damián Viniegra Morales, Francisco A. Baeza Terrazas, María C. Ishida, Daniela S. Gutiérrez-Torres, R. Jesse Saunders, Zuzana Drobná, Rebecca C. Fry, John B. Buse, Dana Loomis, Gonzalo G. García-Vargas, Luz M. Del Razo, Miroslav Stýblo

**Affiliations:** 1 Department of Nutrition, UNC Gillings School of Global Public Health, Chapel Hill, North Carolina, USA; 2 Carolina Population Center, and; 3 Lineberger Cancer Center, University of North Carolina at Chapel Hill, Chapel Hill, North Carolina, USA; 4 Programa de Maestría en Ciencias en Biotecnología, Facultad de Ciencias Químicas, Universidad Autónoma de Chihuahua, Chihuahua, México; 5 Colegio de Médicos Cirujanos y Homeópatas del Estado de Chihuahua, A.C., Mexico; 6 Department of Environmental Sciences and Engineering, UNC Gillings School of Global Public Health, Chapel Hill, North Carolina, USA; 7 Curriculum in Toxicology, University of North Carolina at Chapel Hill, Chapel Hill, North Carolina, USA; 8 Department of Medicine, School of Medicine, University of North Carolina at Chapel Hill, Chapel Hill, North Carolina, USA; 9 International Agency for Research on Cancer, Monographs Section, Lyon Cedex, France; 10 Facultad de Medicina, Universidad Juárez del Estado de Durango, Gómez Palacio, Durango, México; 11 Departamento de Toxicología, Centro de Investigación y de Estudios Avanzados del Instituto Politécnico Nacional, México DF, México

## Abstract

**Background:**

Exposure to arsenic (As) concentrations in drinking water > 150 μg/L has been associated with risk of diabetes and cardiovascular disease, but little is known about the effects of lower exposures.

**Objective:**

This study aimed to examine whether moderate As exposure, or indicators of individual As metabolism at these levels of exposure, are associated with cardiometabolic risk.

**Methods:**

We analyzed cross-sectional associations between arsenic exposure and multiple markers of cardiometabolic risk using drinking-water As measurements and urinary As species data obtained from 1,160 adults in Chihuahua, Mexico, who were recruited in 2008–2013. Fasting blood glucose and lipid levels, the results of an oral glucose tolerance test, and blood pressure were used to characterize cardiometabolic risk. Multivariable logistic, multinomial, and linear regression were used to assess associations between cardiometabolic outcomes and water As or the sum of inorganic and methylated As species in urine.

**Results:**

After multivariable adjustment, concentrations in the second quartile of water As (25.5 to < 47.9 μg/L) and concentrations of total speciated urinary As (< 55.8 μg/L) below the median were significantly associated with elevated triglycerides, high total cholesterol, and diabetes. However, moderate water and urinary As levels were also positively associated with HDL cholesterol. Associations between arsenic exposure and both dysglycemia and triglyceridemia were higher among individuals with higher proportions of dimethylarsenic in urine.

**Conclusions:**

Moderate exposure to As may increase cardiometabolic risk, particularly in individuals with high proportions of urinary dimethylarsenic. In this cohort, As exposure was associated with several markers of increased cardiometabolic risk (diabetes, triglyceridemia, and cholesterolemia), but exposure was also associated with higher rather than lower HDL cholesterol.

**Citation:**

Mendez MA, González-Horta C, Sánchez-Ramírez B, Ballinas-Casarrubias L, Hernández Cerón R, Viniegra Morales D, Baeza Terrazas FA, Ishida MC, Gutiérrez-Torres DS, Saunders RJ, Drobná Z, Fry RC, Buse JB, Loomis D, García-Vargas GG, Del Razo LM, Stýblo M. 2016. Chronic exposure to arsenic and markers of cardiometabolic risk: a cross-sectional study in Chihuahua, Mexico. Environ Health Perspect 124:104–111; http://dx.doi.org/10.1289/ehp.1408742

## Introduction

There is growing evidence that chronic exposure to inorganic As (iAs) may increase the risk of cardiometabolic (CM) disorders, including diabetes mellitus (DM) and cardiovascular diseases (CVD) (Kuo et al. 2013; Maull et al. 2012; Moon et al. 2012). Experimental studies report adverse effects of iAs and its metabolites on mechanisms associated with CM disorders, such as insulin secretion and signaling, lipid metabolism, systemic inflammation, and atherosclerosis (Cheng et al. 2011; Douillet et al. 2013; Druwe et al. 2012; Fu et al. 2010; Lemaire et al. 2011; Muthumani and Prabu 2014; Paul et al. 2007). Recent reviews of the epidemiological literature suggest that exposure to levels of iAs in drinking water > 150 μg As/L may increase the risk of diabetes (Maull et al. 2012) and CVD outcomes (Abhyankar et al. 2012; Moon et al. 2012; Navas-Acien et al. 2005). Evidence of relationships at low to moderate levels of exposure is more limited and equivocal.

To date, few epidemiologic studies have examined associations between moderate iAs exposure and markers of CM risk. Such studies may help to provide insight into the potential role of iAs exposure in the development and progression of CVD and diabetes. A few studies in industrially contaminated areas, or in settings with mean water As concentrations > 150 μg/L, have reported As exposure to be associated with CM markers such as elevated blood pressure and elevated fasting glucose, triglyceride, and low-density lipoprotein (LDL) cholesterol levels (Chen et al. 2012; Karim et al. 2013; Wang et al. 2007). However, there are limited and inconsistent data on associations with CM risk markers, most notably dyslipidemias, at lower As exposures (Abhyankar et al. 2012; Gribble et al. 2012; Jones et al. 2011).

Evidence is also limited regarding the role of iAs metabolism in determining health risks associated with iAs exposure. In humans, iAs is enzymatically methylated to yield methyl- arsenic (MAs) and subsequently dimethylarsenic (DMAs) metabolites that are, along with residual iAs, excreted mainly in urine (Thomas et al. 2007). Urinary As profiles characterized by low percentages of DMAs and high percentages of MAs are thought to indicate a low capacity to methylate iAs. These indicators have been linked to an increased risk of cancer and precancerous skin lesions (Ahsan et al. 2007; Chen et al. 2003a, 2003b; Pierce et al. 2013; Yu et al. 2000). However, the relationship between urinary profiles of iAs metabolites and non-cancerous outcomes remains unclear (Chen et al. 2013b; Del Razo et al. 2011; Huang et al. 2007; Kim et al. 2013; Nizam et al. 2013).

This cross-sectional study explored associations between CM risk and chronic exposure to iAs in a recently established cohort of adult residents of Chihuahua (Mexico) who consume water with a wide range of iAs concentrations. We examined the relationship between iAs in drinking water and urine; we also investigated the relationship between urinary indicators of iAs metabolism and CM risk based on measurements of dysglycemia, including diabetes, dyslipidemia, and blood pressure levels.

## Materials and Methods

*The Chihuahua cohort.* All procedures involving human subjects were approved by institutional review boards at the University of North Carolina at Chapel Hill and Cinvestav-IPN (Centro de Investigación y de Estudios Avanzados del Instituto Politécnico Nacional, Mexico City, Mexico). All participants provided signed informed consent. A total of 1,160 adults (≥ 18 years old) with a minimum 5-year uninterrupted residency in the study area were recruited in household visits between 2008 and 2012. The participation rate was 67%. Other exclusion criteria were pregnancy, self-reported kidney or urinary tract infection (both conditions that affect profiles of iAs metabolites in urine), and potential occupational exposure to As (e.g., working with pesticides or in mines or smelters). Samples of drinking water were obtained from the participants’ households. An interviewer-administered study questionnaire was used to record data on residency, occupation, drinking-water sources and use, smoking, alcohol consumption, and medical history. As described previously (Currier et al. 2014), spot urine and fasting venous blood were collected during a morning medical examination that included an oral glucose tolerance test with blood drawn 2 hr after a 75-g glucose dose. Plasma from both fasting and 2-hr blood samples was stored at –80°C until analysis. Urine samples were aliquoted and immediately frozen. Trained staff obtained measurements of participants’ weight (without shoes and in light clothing) to the nearest 0.1 kg and measurements of their height to the nearest 0.1 cm; this information was used to calculate the body mass index (BMI) of the participants. BMI cutoffs of ≥ 25.0, ≥ 30, and < 18.5 kg/m^2^ were used to define overweight, obese, and underweight individuals, respectively (World Health Organization [WHO] Expert Committee on Physical Status 1995). Participants’ waist circumference was measured at the midpoint between the lowest rib and the iliac crest. Blood pressure was assessed using a manual sphygmomanometer. Three measurements were taken at intervals of at least 1 min, with a 5-min rest before obtaining the first reading; the mean of the last two measurements was used. Participants were seated with their backs supported, feet on the floor, and the arm supported in a horizontal position, with the cuff at the level of the heart.

*Arsenic analyses.* Hydride generation-atomic absorption spectrometry coupled with a cryotrap (HG-CT-AAS) (Hernández-Zavala et al. 2008) was used to determine the concentration of As in drinking water and the concentrations of inorganic and methylated As species in urine. Arsenobetaine, arsenocholine, and arsenosugars cannot be measured using this method. A certified standard reference material, Arsenic Species in Frozen Human Urine (SRM 2669; National Institute of Standards and Technology, Gaithersburg, MD) was used to ensure accuracy. Concentrations of As species measured in SRM 2669 by HG-CT-AAS ranged from 86.7 to 106.4% of the certified values. The limit of detection (LOD) for As in water as well as for As species in urine was 0.01 μg As/L. The concentration of creatinine in urine was determined using a colorimetric assay (Cayman Chemical Company, Ann Arbor, MI). Concentrations of water As and urinary As species below the LOD (1.9% for water As, 1.6% for urinary iAs) were imputed at LOD/2. Total speciated As in urine (tAs) was calculated as the sum of iAs, MAs, and DMAs. The pattern of iAs metabolism was characterized using the percentage of tAs present as DMAs, MAs, and iAs and the ratios of MAs/iAs and DMAs/MAs.

*CM risk markers.* A Prestige 24i Chemistry Analyzer (Tokyo Boeki Medisys Inc., Tokyo, Japan) was used to determine fasting plasma glucose (FPG) and 2-hr plasma glucose (2HPG) concentrations, in addition to triglyceride (TG), total cholesterol (TC), and high-density lipoprotein cholesterol (HDL) concentrations in fasting plasma. Reference human sera (Serodos and Serodos PLUS; Human Diagnostics Worldwide) were used for quality control. LDL was calculated using the Friedewald equation; 28 individuals with measured lipids outside accepted ranges for this approach were excluded from this calculation (Oliveira et al. 2013). Diabetes was classified by FPG ≥ 126 mg/dL, 2HPG ≥ 200 mg/dL, or self-reported diabetes diagnosis or medication use (WHO/International Diabetes Federation 2006). Prediabetes was defined as the absence of diabetes with FPG ≥ 110 mg/dL or 2HPG ≥ 140 mg/dL. Individuals with diabetes or prediabetes were classified as having dysglycemia. Elevated fasting levels of each lipid were defined as plasma TG ≥ 150 mg/dL, TC ≥ 200 mg/dL, and LDL ≥ 130 mg/dL (Miller et al. 2011; National Cholesterol Education Program 2002). Fasting HDL < 40 mg/dL in men and < 50 mg/dL in women were designated as low. Hypertension was defined by systolic blood pressure (SBP) > 140 mmHg, diastolic blood pressure (DBP) > 90 mmHg, or self-reported use of anti-hypertensive medication (Chobanian et al. 2003).

*Statistical analysis.* Associations between iAs exposure and each CM risk marker were analyzed using both categorical and continuous exposure measures. Arsenic concentrations in water and urine, as well as urinary DMAs/MAs and MAs/iAs ratios, were either categorized in quartiles or were natural log–transformed when used as continuous measures to make their distributions more normal. Associations with the percentage of urinary tAs comprised by DMAs, MAs, and iAs were presented as quartiles or dichotomized at the median. Chi-square, ANOVA, and Kruskal–Wallis tests were used as appropriate to determine the significance of differences in subject characteristics by level of iAs exposure.

Multinomial (diabetes and prediabetes vs. neither) or simple logistic regression models (other variables) were used to analyze associations between iAs exposure and each CM risk outcome. To evaluate associations at various exposure doses, both categorical and log-transformed continuous exposure variables were used. Models were adjusted for age, sex, education, ethnicity, smoking, alcohol consumption, waist circumference, BMI, primary source of household drinking water (wells, treatment plants, and other), and self-reported seafood intake in the past week (a potential source of arsenobetaine or arsenosugars). Supplementary models examined the effects of adjusting for log-transformed urinary creatinine concentrations as recommended (Maull et al. 2012) or of normalizing As concentrations by dividing by the concentration of urinary creatinine. Statistical significance of main effects was set at *p* < 0.05 with *p* < 0.10 indicating marginal significance. Product terms (continuous outcomes) or relative excess risk for interaction (categorical outcomes) were calculated to assess interactions (*p* < 0.10) when exploring combined effects of iAs exposure and metabolism (Vanderweele and Knol 2014). Thus, all interactions were evaluated on an additive scale. The primary analysis sample (*n* = 1,090, 94%) excluded individuals with missing data (*n* = 70 for urinary tAs, blood pressure, dysglycemia, or covariates); 37 additional individuals were missing lipid measurements (*n* = 1,053). Water As measurements were unavailable for an additional 52 participants in the analysis sample (*n* = 1,038; 1,004 for lipids). Multiple imputations fit using 10 replicates of chained equations indicated that the results of the complete case analysis did not differ significantly when missing data were imputed (data not shown). All analyses used STATA version 13.1 (StataCorp; College Station, TX).

## Results

*As exposure.* Sociodemographic and anthropometric characteristics of the Chihuahua cohort, as well as data characterizing CM risk prevalence, iAs exposure, and urinary iAs metabolites are provided in Table 1. Concentrations of As in drinking water ranged from below the LOD to 419.8 μg/L, with a median of 47.9 μg/L. A total of 83.3% of the analysis sample exceeded the U.S. Environmental Protection Agency (EPA) and WHO recommended limit of 10 μg As/L, and 75.3% of the analysis sample exceeded the limit in Mexico of 25 μg As/L (Dirección General de Normas 1994; U.S. EPA 2014). Concentrations of total speciated urinary As (tAs) ranged from 0.52 to 491.5 μg/L, with a median of 55.8 μg/L. DMAs was the major metabolite (median 76.8% of tAs), followed by MAs (14.0%) and iAs (8.9%). Urinary tAs (Table 1) and concentrations of each As species increased with increasing concentrations of water As. However, the percentages of MAs and iAs increased with increasing amounts of urinary tAs (see Supplemental Material, Table S1). Water As and urinary tAs were correlated (Spearman’s rho = 0.47).

**Table 1:** Characteristics of the sample by concentration of arsenic in household water.

Characteristic	All participants	Household water arsenic quartiles (μg/L)			
		< 25.5	≥ 25.5 to < 47.9	≥ 47.9 to < 79.0	≥ 79.0
Total *n*	1,038	260	260	259	259
Sociodemographic, lifestyle
Age, years*	45.6 ± 15.9	47.4 ± 16.8	43.4 ± 16.4	44.6 ± 14.5	47.0 ± 15.4
Female	712 (68.6)	180 (69.2)	174 (66.9)	185 (71.4)	173 (66.8)
Higher than primary education*	320 (30.8)	93 (35.8)	106 (40.8)	71 (27.4)	50 (19.3)
Smokes	291 (28.0)	65 (25.0)	71 (27.6)	70 (27.0)	85 (32.8)
Drinks alcohol**	423 (40.8)	90 (34.6)	114 (43.9)	115 (44.4)	104 (40.2)
Recent seafood intake**	260 (25.1)	79 (30.4)	64 (24.6)	64 (24.7)	53 (20.5)
Anthropometric*, *cardiometabolic
Weight status^a^**
Overweight	368 (35.5)	91 (35.0)	88 (33.9)	87 (33.6)	102 (39.4)
Obese	411 (39.6)	92 (35.4)	112 (43.1)	118 (45.6)	89 (34.4)
Waist circumference, cm
Female**	98.8 (13.0)	96.2 (12.0)	100.7 (12.9)	100.1 (14.5)	98.2 (12.1)
Male	96.7 (12.1)	97.7 (11.8)	96.5 (12.0)	97.0 (12.3)	95.6 (12.2)
Dysglycemia^*b*^
Diabetes	183 (17.6)	33 (12.7)	53 (20.4)	47 (18.2)	50 (19.3)
Prediabetes	156 (15.0)	41 (15.8)	37 (14.2)	38 (14.7)	40 (15.4)
Triglycerides ≥ 150 mg/dL*	412 (41.0)	85 (33.5)	104 (41.4)	110 (43.8)	113 (45.6)
Total cholesterol ≥ 200 mg/dL**	234 (23.3)	44 (17.3)	61 (24.3)	67 (26.7)	62 (25.0)
LDL cholesterol ≥ 130 mg/dL^*c*^	160 (16.3)	33 (13.2)	43 (17.4)	45 (18.5)	39 (16.3)
HDL < 40/50 mg/dL**	589 (58.7)	161 (63.4)	151 (60.1)	144 (57.4)	133 (53.6)
Hypertension^*d*^	439 (42.3)	106 (40.8)	106 (40.8)	109 (42.1)	118 (45.6)
Urinary As and dilution markers
Total As^*e*^ (μg/L)*	55.8 (27.1–105)	22.9 (6.5–48.3)	59.0 (35.4–94.3)	62.6 (33.4–101)	96.6 (52.0–150)
DMAs (μg/L)*	42.4 (20.5–77.6)	16.2 (5.1–35.0)	44.0 (26.3–71.7)	62.6 (33.4–101)	96.6 (52.0–150)
MAs (μg/L)*	7.7 (3.2–14.9)	2.7 (0.8–7.3)	8.3 (4.5–13.5)	47.5 (25.9–77.5)	69.5 (38.5–115)
iAs (μg/L)*	5.0 (1.9–10.0)	1.5 (0.5–4.8)	5.7 (2.7–9.3)	8.7 (4.4–14.4)	13.5 (6.0–24.0)
DMAs/MAs	5.5 (4.0–7.4)	5.6 (4.1–7.5)	5.7 (4.2–7.6)	5.4 (4.1–7.4)	5.3 (3.8–7.1)
MAs/iAs	1.6 (1.2–2.1)	1.6 (1.1–2.3)	1.5 (1.1–2.0)	1.6 (1.2–2.1)	1.6 (1.2–2.0)
Percent DMAs	76.8 (70.6–81.5)	76.7 (70.3–81.0)	76.6 (71.3–81.3)	77.2 (71.6–82.8)	76.6 (69.6–81.2)
Percent MAs	14.0 (10.9–17.7)	13.9 (10.8–17.3)	13.5 (10.5–1.37)	14.2 (11.1–17.7)	14.4 (11.2–18.4)
Percent iAs	8.9 (6.4–12.3)	8.8 (6.0–12.9)	9.4 (6.4–12.8)	8.5 (6.4–11.6)	8.9 (6.7–12.1)
Creatinine, mg/dL*	135 (74.7–173)	115 (60–162)	131 (78–190)	140 (80–183)	144 (82–167)
Data are *n* (%), mean ± SD, or median (25th–75th percentile) unless otherwise indicated. One-way analysis of variance (ANOVA), Pearson’s chi-square, or Kruskal–Wallis test for differences across increasing quartiles of water As. Distributions among individuals with household water As, *n *= 1,038 for all variables except LDL (*n *= 980) and other lipids (*n *= 1,004). ^***a***^Weight status: BMI ≥ 25 to < 30 overweight, BMI ≥ 30 obese. ^***b***^Diabetes: fasting plasma glucose (FPG) ≥ 126 mg/dL, 2-hr plasma glucose (2HPG) ≥ 200 mg/dL, or self-reported diabetes diagnosis or medication use. Prediabetes: FPG ≥ 110 to < 126 mg/dL or 2HPG ≥ 140 mg/dL. ^***c***^LDL cholesterol: estimated using the Friedewald equation if triglycerides < 400 mg/dL (Oliveira et al. 2013). ^***d***^Hypertension: SBP > 140 mmHg, DBP > 90 mmHg or anti-hypertensive medication use [medication use reported by *n *= 126 (28.7%) of the hypertensive individuals]. ^***e***^Total speciated urinary arsenic: Σ[dimethylated (DMAs), monomethylated (MAs), and inorganic (iAs) arsenic species]. *p < 0.05; **p < 0.10.					

*Water As and CM risk.* Overall, 18% of the study participants had diabetes (115 of 183 reporting previous diagnosis), and 15% had prediabetes (Table 1). Forty-one percent had elevated TG, 23% had high TC, 16% had high LDL, and 42% had hypertension.

In multivariable-adjusted models (Table 2), drinking water As was associated with several markers of CM risk, including elevated TC and TG, as well as with diabetes (*p* < 0.05 for log water As), with increased risk in the second quartile (≥ 25.5 μg As/L) and no evidence of further increases in risk at higher exposures. However, greater exposures to water As were associated with reduced odds of low HDL, with patterns suggesting a monotonic dose–response curve (*p* < 0.05). Excluding individuals with diabetes (*n* = 183) did not significantly affect relationships with other outcomes associated with water As [adjusted ORs for log-transformed water As were 1.07 (95% CI: 1.01, 1.14) for TG, 1.07 (95% CI: 1.00, 1.15) for TC, and 0.87 (95% CI: 0.82, 0.93) for HDL].

**Table 2:** Household drinking water arsenic concentrations and prevalent cardiometabolic risk outcomes: odds ratios (95% CI).

Cardiometabolic outcome	Water arsenic exposure quartile (μg/L)			ln-Water As (μg/L)
	≥ 25.5 to < 47.9	≥ 47.9 to < 79.0	≥ 79.0	
Dysglycemia^*a*^
Diabetes	2.46 (1.44, 4.21)*	1.74 (1.01, 2.99)*	1.65 (0.97, 2.81)**	1.14 (1.05, 1.25)*
Prediabetes	1.14 (0.68, 1.91)	1.04 (0.62, 1.73)	1.13 (0.68, 1.88)	1.00 (0.94, 1.09)
Triglycerides ≥ 150 mg/dL	1.45 (0.99, 2.14)**	1.53 (1.04, 2.24)*	1.69 (1.15, 2.49)*	1.09 (1.03, 1.15)*
Total Cholesterol ≥ 200 mg/dL	1.75 (1.11, 2.74)*	1.89 (1.21, 2.95)*	1.65 (1.05, 2.59)*	1.08 (1.01, 1.16)*
LDL ≥ 130 mg/dL^*b*^	1.54 (0.92, 2.56)**	1.59 (0.96, 2.65)**	1.35 (0.80, 2.27)	1.04 (0.96, 1.12)
HDL < 40/50 mg/dL	0.78 (0.52, 1.17)	0.63 (0.42, 0.93)*	0.59 (0.40, 0.88)*	0.87 (0.82, 0.93)*
Hypertension^*c*^	1.30 (0.84, 2.00)	1.27 (0.82, 1.94)	1.41 (0.91, 2.17)	1.03 (0.97, 1.10)
Results are derived from multinomial or logistic models adjusted for age, sex, education, smoking status, alcohol consumption, recent seafood intake, weight status, elevated waist circumference, and main water source (well, treatment plant, or other); multinomial models used for diabetes and prediabetes versus neither; logistic models used for other outcomes. ^***a***^Diabetes: fasting plasma glucose (FPG) ≥ 126 mg/dL, 2-hr plasma glucose (2HPG) ≥ 200 mg/dL, or self-reported diabetes diagnosis or medication use. Prediabetes: FPG ≥ 110 to < 126 mg/dL or 2HPG ≥ 140 mg/dL. Normoglycemic individuals (i.e., individuals with no diabetes or prediabetes) are the referent. ^***b***^LDL-cholesterol: estimated using the Friedewald equation if triglycerides < 400 mg/dL (Oliveira et al. 2013). ^***c***^Hypertension: SBP > 140, DBP > 90, or use of anti-hypertensive medication (medication use reported by 27.9% of hypertensive individuals). Normotensive individuals (no stage 1 or 2 hypertension) are the referent. *p < 0.05, **p < 0.10 for odds ratios for elevated versus low cardiometabolic risk associated with increasing water As exposure versus the lowest quartile (< 25.5 μg/L).				

**Figure 1:**
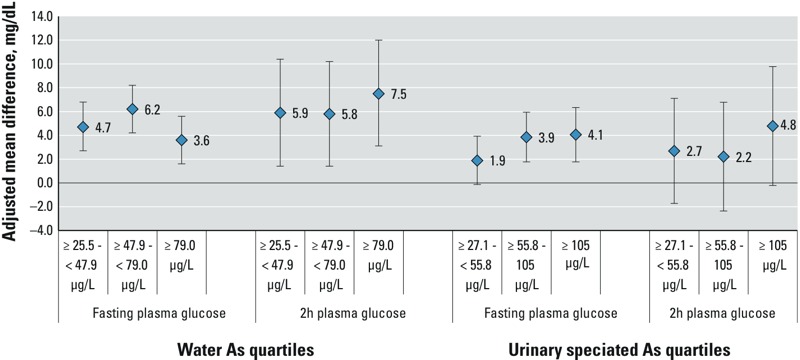
Adjusted mean (95% CI) difference in fasting or 2-hr plasma glucose associated with As exposure among normoglycemic subjects. Adjusted mean (95% CI) difference in glucose measurement for increasing quartiles of water As or total speciated urinary As relative to individuals in the lowest quartile (< 25.5 μg/L for water and < 27.1 μg/L for urine). Estimated from linear regression models including age, sex, education, ethnicity, weight status, waist circumference, smoking status, alcohol consumption, recent seafood intake, and water source (well, treatment plant, or other). Urinary As models were additionally adjusted for urinary creatinine and ≥ median %DMAs, MAs, and iAs. Models excluded individuals with 2-hr plasma glucose > 140 mg/dL, fasting plasma glucose > 110 mg/dL, or diagnosed diabetes. Among normoglycemic subjects: n in each quartile of water As 1 = 186, 2 = 170, 3 = 174, 4 = 169; of urinary As: 1 = 195, 2 = 181, 3 = 186, 4 = 170.

Associations with continuous CM measurements are shown in the Supplemental Material, Table S2. Although water As was not associated with prediabetes (Table 2), increasing exposure was associated with elevated mean FPG and 2HPG among individuals not using diabetes medications and among individuals without diabetes (see Supplemental Material, Table S2). After multivariable adjustment, increasing concentrations of water As were also associated with significant increases in mean FPG and 2HPG among fully normoglycemic participants (i.e., individuals without either diabetes or prediabetes; Figure 1). Consistent with the categorical outcomes, after adjustment, increasing concentrations of water As were associated with increases in mean TG and TC and decreases in mean HDL, but not with mean LDL. Water As was not associated with mean DBP; the association with SBP was attenuated when individuals with diabetes were excluded (see Supplemental Material, Table S2).

*Urinary tAs and CM risk.* Urinary tAs concentrations were associated with multiple markers of CM risk. Similarly to the findings for water As, increasing concentrations of urinary tAs were associated with increased odds of diabetes and elevated TG, and there was evidence of increased risk at moderate concentrations (≥ 27.1 to < 55.8 μg/L) (Table 3). The highest quartile of tAs (≥ 105 μg/L) was associated with elevated TC (*p* < 0.05). There were, however, reduced odds of low HDL associated with log tAs (*p* < 0.05). Additional adjustment for urinary creatinine and urinary tAs metabolite composition (Table 3) tended to strengthen the magnitude of the associations. Similarly to water As, urinary tAs was not associated with prediabetes, but it was associated with significant increases in mean FPG among normoglycemic individuals in multivariable adjusted models (Figure 1); the highest quartile of tAs was also associated with mean increases in 2HPG. Excluding individuals with diagnosed diabetes did not meaningfully influence relationships between urinary tAs and other outcomes [adjusted ORs for the highest vs. lowest quartiles: 1.71 (95% CI: 1.08, 2.71) for high TG, 2.14 (95% CI: 1.26, 3.62) for high TC, and 0.71 (95% CI: 0.44, 1.12) for low HDL].

**Table 3:** Total speciated urinary As and prevalent cardiometabolic risk: odds ratios (95% CIs).

Cardiometabolic outcome	Total urinary speciated arsenic quartiles (μg/L)			ln-Total urinary As (μg/L)
	≥ 27.1 to < 55.8	≥ 55.8 to <105.0	≥ 105.0	
*n*	272	273	272	—
Multivariable adjusted
Dysglycemia^*a*^
Diabetes	1.57 (0.94, 2.63)**	1.56 (0.92, 2.65)**	1.99 (1.19, 3.33)*	1.29 (1.09, 1.53)*
Prediabetes	0.92 (0.56, 1.53)	1.21 (0.74, 1.98)	1.15 (0.69, 1.92)	1.04 (0.89, 1.23)
Triglycerides ≥ 150 mg/dL	1.39 (0.95, 2.02)**	1.47 (1.01, 2.13)*	1.80 (1.23, 2.64)*	1.23 (1.08, 1.39)*
Cholesterol ≥ 200 mg/dL	1.15 (0.74, 1.78)	1.35 (0.88, 2.07)	1.54 (1.00, 2.38)*	1.15 (1.00, 1.33)*
LDL ≥ 130 mg/dL^*b*^	0.99 (0.60, 1.62)	1.22 (0.75, 1.99)	1.25 (0.76, 2.05)	1.09 (0.93, 1.28)
HDL < 40/50 mg/dL	0.94 (0.64, 1.27)	1.09 (0.74, 1.60)	0.82 (0.56, 1.21)	0.82 (0.72, 0.93)*
Hypertension^*c*^	0.67 (0.44, 1.01)*	0.60 (0.40, 0.92)*	0.77 (0.50, 1.17)	0.93 (0.72, 1.07)
Additionally adjusted for creatinine and elevated %DMAs, MAs, iAs in urine
Dysglycemia
Diabetes	1.76 (1.03, 3.02)*	1.98 (1.12, 3.50)*	2.78 (1.55, 5.00)*	1.45 (1.19, 1.77)*
Prediabetes	0.89 (0.53, 1.51)	1.22 (0.72, 2.08)	1.16 (0.65, 2.04)	1.04 (0.86, 1.25)
Triglycerides ≥ 150 mg/dL	1.41 (0.95, 2.08)**	1.55 (1.04, 2.32)*	1.96 (1.28, 3.00)*	1.25 (1.08, 1.44)*
Cholesterol ≥ 200 mg/dL	1.25 (0.80, 1.96)	1.56 (0.98, 2.47)**	1.89 (1.16, 3.06)*	1.22 (1.04, 1.44)*
LDL ≥ 130 mg/dL	1.08 (0.65, 1.81)	1.42 (0.84, 2.40)	1.54 (0.88, 2.69)	1.16 (0.96, 1.40)
HDL < 40/50 mg/dL	0.92 (0.62, 1.36)	1.09 (0.73, 1.64)	0.81 (0.52, 1.25)	0.87 (0.77, 0.99)*
Hypertension^*c*^	0.74 (0.48, 1.14)	0.73 (0.47, 1.14)	1.02 (0.64, 1.61)	1.03 (0.89, 1.20)
Results are derived from multinomial or logistic models adjusted for age, sex, education, smoking status, alcohol consumption, recent seafood intake, weight status, elevated waist circumference, and main water source (well, treatment plant, or other); multinomial model used for diabetes and prediabetes versus neither; logistic models used for other outcomes. ln-Transformed urinary creatinine and > median %DMAs, MAs, and iAs in urine additionally included in models as indicated. ^***a***^Diabetes: fasting plasma glucose (FPG) ≥ 126 mg/dL, 2-hr plasma glucose (2HPG) ≥ 200 mg/dL, or self-reported diabetes diagnosis or medication use. Prediabetes: FPG ≥ 110 to < 126 mg/dL or 2HPG ≥ 140 mg/dL. Normoglycemic individuals (i.e., individuals with no diabetes or prediabetes) are the referent. ^***b***^LDL-cholesterol: estimated using the Friedewald equation if triglycerides < 400 mg/dL (Oliveira et al. 2013). ^***c***^Hypertension: SBP > 140, DBP > 90, or use of anti-hypertensive medication (medication use reported by 27.9% of hypertensive individuals). Normotensive individuals (no stage 1 or 2 hypertension) are the referent. *p < 0.05, **p < 0.10 for odds ratios for elevated vs. low cardiometabolic risk associated with increasing water As exposure vs. referent of < 27.5 μg/L (*n *= 273).				

The relationships with continuous CM markers after multivariable adjustment (see Supplemental Material, Table S2) were similar to those with categorical outcomes, with urinary tAs positively associated with TG, TC, HDL, and FPG, as well as with 2HPG in subjects not using medications to control the levels of those markers. Among individuals without diabetes, associations with 2HPG were attenuated. Urinary tAs was not associated with SBP or DBP even when normalized to creatinine [coefficients in individuals not using anti-hypertensive medication: 1.19 (95% CI: –0.29, 2.66) *p* = 0.12 for SBP; 0.02 (95% CI: 0.88, 0.93) *p* = 0.95 for DBP].

**Figure 2:**
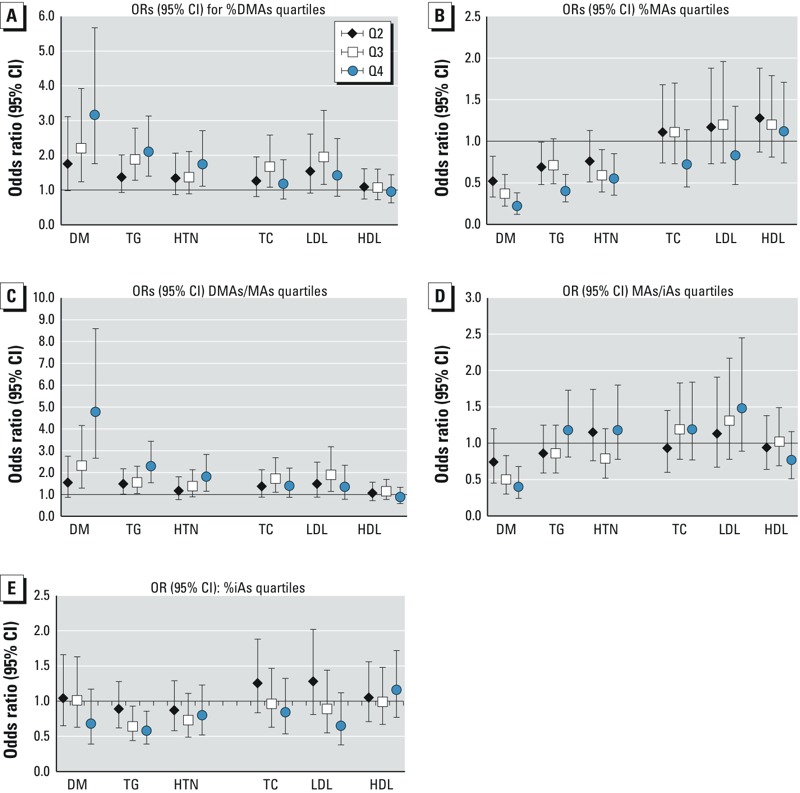
Associations between urinary As metabolism indicators and cardiometabolic risk. Odds ratios (95% CIs) for elevated cardiometabolic risk associated with increasing quartiles of urinary iAs metabolism indicators from multinomial or logistic models adjusted for total speciated urinary arsenic, as well as age, sex, education, ethnicity, weight status, waist circumference, smoking status, alcohol consumption, recent seafood intake, and water source (well, treatment plant, or other). *n *= 1,090 Adults. Cardiometabolic markers: DM, diabetes mellitus; HDL, high-density lipoprotein cholesterol; HTN, hypertension; LDL, low-density lipoprotein cholesterol; TC, total cholesterol; TG, triglycerides. Urinary As indicators: DMAs, dimethylarsenic; iAs, inorganic As; MAs, methylarsenic. Cardiometabolic outcomes defined as follows: DM, fasting plasma glucose ≥ 126 mg/dL, 2-hr plasma glucose ≥ 200 mg/dL, or self-reported diabetes diagnosis or medication use; elevated TC ≥ 200 mg/dL; elevated TG ≥ 200 mg/dL; elevated LDL ≥ 130 mg/dL; low HDL = < 40 mg/dL; hypertension SBP > 140 mmHg, DBP > 90 mmHg or anti-hypertensive medication use. Quartile markers (1st = referent): 2nd, black diamond; 3rd, white square; 4th, blue circle. Quartile cutoffs for urinary As metabolism indicators defined as follows: (*A*) %DMAs = < 70.65, 70.65 to < 76.78, 76.78 to < 81.52, ≥ 81.52; (*B*) %MAs = < 10.90, 10.90 to < 14.0, 14.0 to < 17.66, ≥ 17.66; (*C*) DMAs/MAs = < 4.05, 4.05 to < 5.47, 5.49 to < 7.38, ≥ 7.38; (*D*) MAs/iAs = < 1.185, 1.185 to < 1.576, 1.576 to < 2.11, ≥ 2.11; (*E*) %iAs = < 6.389, 6.389 to < 8.873, 8.873 to < 12.270, ≥ 12.270.

*iAs metabolism and CM risk markers.* Higher %DMAs and DMAs/MAs in urine were associated with increased odds of diabetes, elevated TG, and hypertension (Figure 2). The relationships between these indicators and other CM outcomes were non-linear and weak. Conversely, an elevated %MAs was associated with reduced odds of diabetes, elevated TG, and hypertension. Similarly to %MAs, a high MAs/iAs ratio was negatively associated with diabetes. Associations of this ratio with other outcomes did not reach significance (*p* < 0.05), but in contrast to diabetes, such associations generally suggested weak increases in risk. A high %iAs was associated with reduced odds of elevated TG [adjusted OR for the highest vs. lowest quartiles: 0.58 (95% CI: 0.39, 1.86)]; other associations were weaker and were not significant.

**Figure 3:**
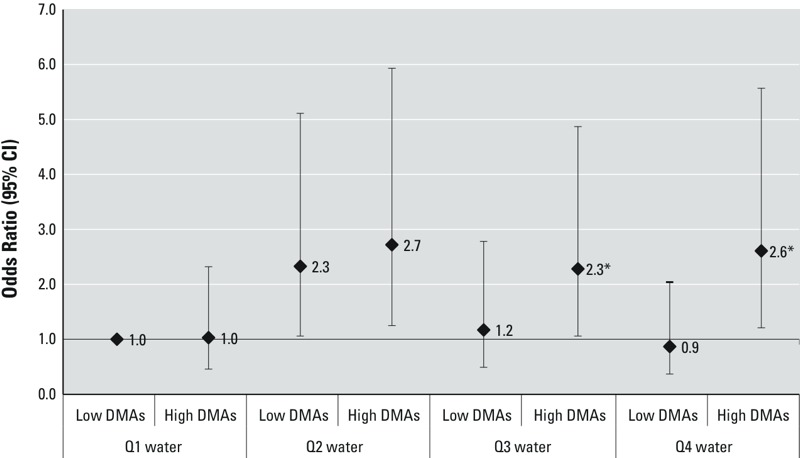
Water As and odds of prevalent diabetes in subjects with vs. without elevated % urinary DMAs. ORs (95% CIs) for prevalent diabetes associated with household water As categories (< 25, 25 to < 50, 50 to < 100, and ≥ 100 μg/L) in subjects with proportions of DMAs defined as low versus high based on the median of 76.6%. The referent group for all ORs is subjects with %DMAs below the median in the lowest quartile of water As. The results are derived from multinomial models adjusted for age, sex, smoking status, alcohol consumption, BMI, elevated waist circumference, and main water source (well, treatment plant, or other). *p < 0.10 for additive interaction (relative excess risk for interaction) for the joint effect of water As and high %DMAs.

*iAs metabolism, iAs exposure, and CM risk markers.* We also examined joint effects of iAs metabolism and iAs exposure to assess whether associations between CM risk markers and As in drinking water varied depending on profiles of iAs metabolites in urine. For subjects with high levels of exposure to water As, the odds of diabetes (Figure 3) and of elevated TG (see Supplemental Material, Table S3) were significantly increased when individuals had not only higher exposure but also elevated %DMAs in urine (interaction *p* < 0.10). For example, the adjusted OR for diabetes associated with being in the highest versus the lowest quartile of water As was 2.61 (95% CI: 1.22, 5.57) for participants with elevated %DMAs, but it was 0.87 (95% CI: 0.37, 2.04) for participants with low DMAs. Similarly, an elevated %DMAs increased the odds of elevated TG associated with elevated concentrations of urinary tAs (interaction *p* < 0.10; see Supplemental Material, Table S3). The adjusted OR for the association between the highest versus the lowest quartiles of water As and high TG was 3.31 (95% CI: 1.89, 5.78) versus 1.18 (95% CI: 0.66, 2.08). Using continuous CM outcomes, among subjects without diabetes, the multivariable-adjusted mean increases in 2HPG and triglycerides associated with water As were significantly larger when %DMAs was elevated (interaction *p* < 0.10) (see Supplemental Material, Table S2). Increases in mean FPG, 2HPG, and triglycerides associated with elevated urinary tAs were also stronger among individuals with elevated %DMAs than among those with decreased %DMAs (interaction *p* < 0.10 for all) (see Supplemental Material, Table S2).

## Discussion

In this study, both moderate exposure to As in drinking water and modest concentrations of speciated urinary As were associated with several CM risk markers. Water As concentrations ≥ 25.5 μg/L were associated with increased odds of diabetes and elevated plasma TG and TC. Similar concentrations of urinary tAs (≥ 27.1 μg/L) were also associated with diabetes and elevated TG, with higher levels (≥ 105.0 μg/L) associated with high TC. Although neither water As nor urinary tAs was associated with increased odds of prediabetes (Tables 2 and 3), both were positively associated with increases in mean FPG and 2HPG among normoglycemic individuals (Figure 1). Unexpectedly, we found that iAs exposure was associated with decreased odds of low HDL. Associations with hypertension and blood pressure measures, or with LDL, were weak or null within the range of exposures in the present study.

When we used urinary As profiles to characterize capacity to metabolize iAs, we found that an elevated %DMAs—or a decreased %MAs—was associated with increased odds of diabetes, elevated TG, and hypertension. Moreover, the increased odds of diabetes and elevated TG associated with increased concentrations of water As were stronger among individuals with an elevated %DMAs. This finding suggests that individuals with patterns of metabolism characterized by this marker may have increased susceptibility to adverse health outcomes associated with As exposure. Similarly, for urinary tAs, individuals with an elevated %DMAs had significantly higher mean increases in fasting and 2-hr glucose, and fasting TG and TC than individuals with decreased %DMAs.

Elevated %DMAs and decreased %MAs, as well as elevated DMAs/MAs and MAs/iAs ratios, have been proposed as indicators of efficient enzymatic methylation of iAs (Del Razo et al. 1997; Tseng 2007). Several studies in settings with high iAs exposure have reported a high %MAs to be associated with markers of increased CM risk (Chen et al. 2013a, 2013b; Li et al. 2013a). Results from our independent population-based study in the Zimapán and Lagunera regions of Mexico suggest that the positive association with urinary DMAs species may be at least partly attributable to elevated urinary concentrations of the toxic trivalent form of DMAs, DMAs^III^ (Del Razo et al. 2011). Notably, consistent with our findings, several recent studies also found a high DMAs/MAs ratio, a high %DMAs, or a decreased %MAs in urine to be associated with an increased risk of diabetes, metabolic syndrome, or individual CM risk markers (Chen et al. 2012; Del Razo et al. 2011; Kim et al. 2013; Moon et al. 2013; Nizam et al. 2013). Additional research is needed to assess the extent to which the toxic trivalent metabolites DMAs^III^ and MAs^III^ may influence CM risk associated with iAs exposure (Calatayud et al. 2014; Navas-Acien et al. 2006; Stýblo et al. 2002).

In line with our findings, several recent prospective studies have found varying levels of iAs exposure—including median urinary As concentrations ≤ 20 μg/L as well as > 200 μg/L—to be associated with increased morbidity and mortality from CVD outcomes, including ischemic heart disease, stroke, and diabetes (Chen et al. 2011, 2013b; James et al. 2013; Kim et al. 2013; Moon et al. 2012, 2013; Navas-Acien et al. 2008, 2009). Previous cross-sectional studies in settings with high levels of As exposure reported elevated levels of As in water or hair to be associated not only with elevated TC, TG, and fasting glucose but also with elevated LDL, decreased HDL, elevations in both SBP and DBP, and systemic inflammation (Chen et al. 2012; Karim et al. 2013; Wang et al. 2007). These studies suggest the possibility of additional or more severe CM effects at higher levels of exposure.

The present study found a high proportion of DMAs to be associated with hypertension. However, the associations between iAs exposure and hypertension were weak and were not significant, perhaps in part due to the moderate exposure levels. Notably, associations with hypertension have been heterogeneous in areas with water As < 400 μg/L, albeit consistent at higher levels of exposure (Abhyankar et al. 2012; Islam et al. 2012; Jones et al. 2011). Normalizing versus adjusting for creatinine slightly strengthened the associations with urinary As; however, studies reporting associations with hypertension using this metric (Li et al. 2013b) have also had considerably higher exposures than those in this population: median 136 μg versus 47.4 μg As/g creatinine. Future research should assess whether moderate exposure may be more closely linked to alternative indicators of vascular function (Kunrath et al. 2013; Li et al. 2013b; Wu et al. 2012) or may vary in genetically or nutritionally vulnerable subgroups with varying iAs metabolism (Chen et al. 2007).

Despite increases in mean 2HPG and FPG associated with iAs exposure among normoglycemic individuals (Figure 1), iAs exposure was not associated with prediabetes. It is possible that the cutoffs used to characterize prediabetes were not sufficiently sensitive in our population, that iAs exposure may promote rapid progression to more severe disease, or that associations with glucose measurements are not causal.

Although additional prospective studies are needed to evaluate these relationships, laboratory studies suggest that iAs or its metabolites may inhibit insulin secretion or signaling (Douillet et al. 2013; Fu et al. 2010; Paul et al. 2007), alter lipid metabolism (Cheng et al. 2011; Hossain et al. 2013; Muthumani and Prabu 2014), and generate proinflammatory responses (Calatayud et al. 2014; Druwe et al. 2012). Increases in TC, LDL, and TG—along with decreases in HDL—have been observed in rodents treated with iAs (Muthumani and Prabu 2014), in addition to increases in hypertension, cardiac hypertrophy, and atherosclerosis (Cheng et al. 2011; Lemaire et al. 2011; Sanchez-Soria et al. 2012). The unexpected association with HDL requires further study in diverse populations and in laboratory settings. It is important to note that large disparities in the prevalence, predictors, and health consequences of low HDL have been described in Mexican and other Hispanic populations compared with populations of European descent (Aguilar-Salinas et al. 2001; Morales et al. 2014; Paramsothy et al. 2010; Salas et al. 2014).

Despite the limitations inherent in a cross-sectional design, the findings from this study are largely consistent with those from experimental research and from several smaller epidemiological studies in high-exposure settings. Moreover, although personal exposure was characterized on the basis of a single urine sample, the findings for water As and urinary tAs were largely consistent. Few previous studies have provided comparisons of urinary and water As measures or have used urinary indicators to assess how metabolism may modify the health effects of environmental exposure through drinking water contaminated by As (Chen et al. 2013a, 2013b). The consistency of unadjusted and multivariable-adjusted results, as well as those of sensitivity analyses excluding individuals previously diagnosed with hypertension or diabetes who may have adjusted behaviors such as water consumption, also supports the possibility of a causal relationship. Moreover, the close resemblance of the high prevalence of obesity and cardiometabolic risk reported herein to that reported for the general population of Mexico (Barquera et al. 2009; Salas et al. 2014; Villalpando et al. 2010) does not suggest selectivity in our cohort with respect to these outcomes. Studies in such settings may help provide a more complete understanding of how iAs exposure may influence cardiometabolic risk because many previous studies of these relationships have been conducted in settings such as Bangladesh, where the prevalence of obesity and CM disorders is relatively low (Chen et al. 2013b; Pan et al. 2013).

## Conclusion

In summary, the results of this study fill a gap in current knowledge by suggesting potential CM risks associated with chronic exposure to As at levels < 100 μg/L in drinking water (Maull et al. 2012; Moon et al. 2013). Associations with measurements of dyslipidemia, which have been infrequently studied to date, warrant further study, given that the implications of our results for health risks were inconsistent for HDL, LDL, and triglycerides. Studies that incorporate measurements of specific lipid fractions and particles may be better able to evaluate the health risks of any association with iAs exposure (Genest 2008; Vickers and Remaley 2014). Our findings also suggest that iAs metabolism may influence the extent to which environmental exposure to iAs adversely affects the risk of CM impairment. Studies that measure trivalent and pentavalent urinary As species are needed to better understand the impact of metabolism on the health risks associated with iAs exposure.

## Supplemental Material

(369 KB) PDF
