# Supplementary material for: Chronic Exposure to Arsenic and Markers of Cardiometabolic Risk: A Cross-Sectional Study in Chihuahua, Mexico
Source: Environ Health Perspect. 2015 Jun 12;124(1):104–11. doi: 10.1289/ehp.1408742 (PMC4710594; doi:10.1289/ehp.1408742)
Supplement: (369 KB) PDF [file ehp.1408742.s001.acco.pdf]

Environ Health Perspect
DOI: 10.1289/ehp.1408742

Note to Readers: *EHP* strives to ensure that all journal content is accessible to all readers. However, some figures and Supplemental Material published in *EHP* articles may not conform to 508 standards due to the complexity of the information being presented. If you need assistance accessing journal content, please contact ehp508@niehs.nih.gov. Our staff will work with you to assess and meet your accessibility needs within 3 working days.

Supplemental Material

Chronic Exposure to Arsenic and Markers of Cardiometabolic Risk—A Cross-Sectional Study in Chihuahua, Mexico

Michelle A. Mendez, Carmen González-Horta, Blanca Sánchez-Ramírez, Lourdes Ballinas-Casarrubias, Roberto Hernández Cerón, Damián Viniegra Morales, Francisco A. Baeza Terrazas, María C. Ishida, Daniela S. Gutiérrez-Torres, R. Jesse Saunders, Zuzana Drobná, Rebecca C. Fry, John B. Buse, Dana Loomis, Gonzalo G. García-Vargas, Luz M. Del Razo, and Miroslav Stýblo

Table of Contents

Table S1. Urinary As metabolites by quartile of total speciated urinary As: Median (25th-75th percentile).

Table S2. Associations between water and urinary arsenic and continuous indicators of cardiometabolic risk: Coefficient 95% CI.

Table S3. Joint effect of water arsenic combined with arsenic metabolism indicators on cardiometabolic risk: Odds ratios (95% CI).

Table S1. Urinary As metabolites by quartile of total speciated urinary As: Median (25th-75th percentile).

	All participants[1]	Total speciated urinary As quartile (µg/L)			
		**<27.1**	**≥27.1-<55.8**	**≥55.8-105**	**≥105**
DMAs *(µg/L)*‡	42.2 (20.5-77.6)	9.9 (4.9-15.9)	30.4 (25.6-36.5)	55.6 (48.5-67.8)	113.1 (91.1-143)
MAs *(µg/L)*‡	7.7 (3.3-14.9)	1.7(0.8-2.6)	5.4 (4.1-7.3)	11.0 (8.5-14.0)	21.6 (16.2-30.0)
iAs *(µg/L)*‡	5.0 (1.9-10.0)	0.7 (0.4-1.6)	3.5 (2.3-4.9)	7.1 (5.2-9.2)	14.3 (10.3-21.3)
DMAs/MAs ratio‡	5.5 (4.0-7.4)	5.8 (4.3-7.9)	5.8 (4.3-7.6)	5.2 (3.9-7.3)	5.1 (3.8-6.8)
MAs/iAs ratio‡	1.6 (1.2-2.1)	1.7 (1.2-2.9)	1.6 (1.2-2.2)	1.6 (1.2-1.9)	1.5 (1.2-1.9)
% DMAs‡	76.8 (70.6-81.5)	78.5 (72.4-83.2)	77.2 (72.4-82.4)	76.0 (70.1-80.8)	75.6 (69.4-80.0)
% MAs‡	14.0 (10.9-17.7)	13.5 (10.3-16.9)	13.4 (10.7-16.9)	14.5 (11.1-18.4)	14.7 (11.7-18.2)
% iAs‡	8.9 (6.4-12.3)	7.9 (4.9-12.3)	8.6 (6.1-12.0)	9.1 (7.0-12.2)	9.7 (7.2-13.0)
Creatinine *(µg/L)* ‡	135 (74.8-173)	79 (44-135)	121 (69-160)	143 (95-183)	163 (132-213)

[1]N=1090 participants with measures of speciated urinary As in the primary analysis sample.

‡ Kruskall-Wallis test P<0.05

Table S2. Associations between water and urinary arsenic and continuous indicators of cardiometabolic risk: Coefficient 95% CI.

		**Water As**					**Urinary tAs**			
**Cardiometabolic risk marker**	**N**	**Ln Water As** *(age, gender)*		**Ln Water As** *(multivariable)*		**N**	**Ln Urinary tAs** *(age and gender)*		**Ln Urinary tAs** *(multivariable)*	
		**β**	**(95% CI)**	**B**	**(95% CI)**		**β**	**(95% CI)**	**β**	**(95% CI)**
Subjects not on medication for condition of interest[a]										
Fasting glucose, mg/dL	957	0.92	(0.17, 1.67)*	0.93	(0.17, 1.69)*	1003	2.21	(0.59, 3.83)*	3.15	(1.30, 5.00)*
2-hour glucose, mg/dL	957	1.54	(0.18, 2.90)*	1.41	(0.05, 2.78)*	1003	3.58	(0.56, 6.61)*	4.13	(0.72, 7.53)*
Triglycerides, mg/dL	1004	4.57	(2.35, 6.78)*	4.05	(1.86, 6.25)*	1053	9.39	(4.51, 14.28)*	8.14	(2.63, 13.65)*
Cholesterol, mg/dL	1004	2.15	(1.00, 3.30)*	2.21	(1.06, 3.37)*	1053	3.41	(0.91, 5.91)*	4.74	(1.85, 7.63)*
LDL, mg/dL	980	0.60	(-0.37, 1.57)	0.68	(-0.30, 1.66)	1026	0.58	(-1.51, 2.67)	1.58	(-0.85, 4.01)
HDL, mg/dL	1004	0.85	(0.56, 1.14)*	0.96	(0.67, 1.25)*	1053	0.78	(0.15, 1.41)*	1.29	(0.57, 2.02)*
SBP, mm Hg	912	0.67	(0.16, 1.18)*	0.60	(0.11, 1.09)*	960	-0.43	(-1.53, 0.66)	0.48	(-0.72, 1.68)
DBP, mm Hg	912	0.15	(-0.18, 0.47)	0.09	(-0.22, 0.40)	960	-0.40	(-1.08, 0.29)	0.02	(-0.72, 0.76)
No diabetes[b]										
Fasting glucose, mg/dL	855	0.29	(-0.02, 0.60)**	0.34	(0.03, 0.65)*	897	0.81	(-0.14, 1.48)*	0.87	(0.10, 1.63)*
2-hour glucose, mg/dL	855	0.62	(-0.18, 1.42)	0.60	(-0.20, 1.39)	897	0.62	(-1.20, 2.44)	1.39	(-0.58, 3.35)
Triglycerides, mg/dL	824	3.58	(1.32, 5.84)*	3.44	(1.20, 5.67)*	863	7.65	(2.76, 12.53)*	5.80	(0.29, 11.30)*
Cholesterol, mg/dL	824	1.95	(0.77, 3.14)*	2.10	(0.91, 3.29)*	863	2.51	(-0.11, 5.13)	3.26	(0.23, 6.29)*
LDL, mg/dL	808	0.58	(-0.41, 1.57)	0.71	(-0.29, 1.71)	846	0.03	(-2.17, 2.22)	0.67	(-1.88, 3.22)
HDL, mg/dL	824	0.87	(0.57, 1.18)*	0.94	(0.63, 1.25)*	863	0.82	(0.14, 1.50)*	1.30	(0.52, 2.09)*
SBP, mm Hg	772	0.44	(-0.07, 0.96)**	0.45	(-0.04, 0.95)**	813	-0.56	(-1.68, 0.56)	0.61	(-0.60, 1.83)
DBP, mm Hg	772	0.06	(-0.28, 0.40)	0.05	(-0.27, 0.38)	813	-0.49	(-1.22, 0.23)	0.00	(-0.78, 0.79)
No diabetes, by %DMAs										
Fasting glucose, mg/dL										
%DMAs ≤median[#]	452	0.11	(-0.28, 0.51)	0.20	(-0.20, 0.60)	452	0.42	(-0.40, 1.23)	0.51	(-0.43, 1.45)
%DMAs >median	403	0.49	(0.01, 0.97)*	0.46	(-0.03, 0.94)	403	1.64	(0.53, 2.75)*	1.22	(-0.09, 2.53)**
2-hour glucose, mg/dL										
%DMAs ≤median	452	-0.22	(-1.31, 0.87)	-0.04	(-1.14, 1.05)	452	-0.87	(-3.21, 1.46)	-0.47	(-3.03, 2.09)
%DMAs >median[#,†]	403	1.49	(0.33, 2.66)*	1.40	(0.22, 2.57)*	403	3.90	(1.12, 6.68)*	3.92	(0.76, 7.07)*
Triglycerides, mg/dL										
%DMAs ≤median[#,†]	369	2.57	(-0.68, 5.82)	2.74	(-0.47, 5.94)**	390	3.34	(-3.37, 10.06)	3.02	(-3.64, 9.69)
%DMAs >median	301	5.19	(1.77, 8.62)*	4.73	(1.21, 8.26)*	310	14.73	(6.63, 22.83)*	13.13	(4.65, 21.60)*

Cardiometabolic risk marker	Water As N	Ln Water As (age, gender) β	(95% CI)	Ln Water As (multivariable) β	(95% CI)	Urinary tAs N	Ln Urinary As (age and gender) β	(95% CI)	Ln Urinary As (multivariable) β	(95% CI)
Cholesterol, mg/dL										
%DMAs ≤median[#]	369	2.20	(0.47, 3.92)*	2.32	(0.55, 4.09)*	390	0.76	(-2.85, 4.38)	0.97	(-2.76, 4.70)
%DMAs >median	301	2.51	(0.58, 4.45)*	2.54	(0.58, 4.51)*	310	4.45	(-0.29, 9.20)**	3.51	(-1.42, 8.43)
LDL, mg/dL										
%DMAs ≤median	365	0.75	(-0.72, 2.21)	0.84	(-0.67, 2.34)	385	0.39	(-3.45, 2.68)	-0.31	(-3.49, 2.86)
%DMAs >median	295	0.81	(-0.87, 2.48)	0.81	(-0.91, 2.53)	304	0.10	(-4.02, 4.21)	-0.70	(-4.99, 3.59)
HDL, mg/dL										
%DMAs ≤median[#]	369	1.07	(0.55, 1.59)*	1.12	(0.59, 1.64)*	390	0.23	(-0.87, 1.33)	0.45	(-0.66, 1.56)
%DMAs >median	301	0.83	(0.39, 1.28)*	0.93	(0.47, 1.40)*	310	1.47	(0.40, 2.55)*	1.66	(0.54, 2.80)*
SBP, mm Hg										
%DMAs ≤median[†]	361	0.12	(-0.63, 0.86)	0.05	(-0.67, 0.76)	383	-1.04	(-2.59, 0.51)	-1.09	(-2.59, 0.41)
%DMAs >median	278	0.93	(0.11, 1.75)*	0.66	(-0.14, 1.47)	289	0.41	(-1.57, 2.38)	0.15	(-1.81, 2.11)
DBP, mm Hg										
%DMAs ≤median[†]	361	-0.42	(-0.95, 0.10)	-0.47	(-0.98, 0.03)**	383	-0.77	(-1.85, 0.31)	-0.96	(-2.02, 0.09)*
%DMAs >median	278	0.56	(0.04, 1.07)*	0.40	(-0.09, 0.90)	289	-0.01	(-1.24, 1.21)	-0.19	(-1.38, 1.01)

*P<0.05 **P<0.10 for coefficient (95% CI) from linear models adjusted for age and gender; multivariable models also include education, smoking status, alcohol consumer, recent seafood intake, weight status, elevated waist circumference and water source. Urinary As models additionally adjusted for log-transformed urinary creatinine and > median % DMAs, Mas, and iAs in urine. Urinary As = Σ[dimethylated (DMAs), mono-methylated (Mas) and inorganic (iAs) species].

[#]Ln urinary As by elevated %DMAs (>76.6%) interaction P<0.10 in both age/gender- and multivariable-adjusted models, evaluated using product terms.

[†]Ln water As by elevated %DMAs (>76.6%) interaction P<0.10 in both age/gender- and multivariable-adjusted models, evaluated using product terms.

[a]*Exclusions:* Fasting and 2-hour glucose were analyzed among individuals not reporting use of diabetes medication; SBP and DBP analyzed among individuals not reporting use of hypertensive medication. Diabetes and hypertension medicine reported to be used by n=87 and 130 individuals respectively in the primary analysis sample (n=81 and 126 with water As measures). [b]*No diabetes:* Excludes individuals with fasting plasma glucose ≥126 mg/dL, 2h plasma glucose ≥200 mg/dL, or self-reported diabetes diagnosis.

Table S3. Joint effect of water arsenic combined with arsenic metabolism indicators on cardiometabolic risk: Odds ratios (95% CI).

Cardiometabolic Outcome	As metabolism Indicator	Household water arsenic concentration quartile (μg/L)			
		<25.5	≥25.5-<47.9	≥47.9-<79.0	≥79.0
Dysglycemia[a]	%DMAs[b]				
Diabetes	Low	1.00	2.32 (1.06-5.11)*	**1.17 (0.49-2.78)**	**0.87 (0.37-2.04)**
	High	1.03 (0.46-2.31)	2.72 (1.25-5.93)*	**2.28 (1.06-4.88)***,#	**2.61 (1.22-5.57)***,#
Prediabetes	Low	1.00	0.59 (0.26-1.36)	1.14 (0.55-2.35)	0.88 (0.42-1.84)
	High	0.92 (0.45-1.86)	1.62 (0.81-3.22)	0.90 (0.43-1.87)	1.34 (0.66-2.73)
Triglycerides ≥150 mg/dL	Low	1.00	1.49 (0.85-2.61)	**1.58 (0.90-2.78)**	**1.18 (0.66-2.08)**
	High	1.43 (0.83-2.49)	**2.01** (1.16-3.47)*,#	**2.12 (1.22-3.66)***,#	**3.31 (1.89-5.78)***,#
Cholesterol ≥200 mg/dL	Low	1.00	1.47 (0.60-2.29)	2.19 (1.14-4.19)	1.52 (0.78-2.95)
	High	1.18 (0.59-2.28)	2.34 (0.98-3.61)**	1.95 (1.03-3.70)*	2.09 (1.10-3.96)*
LDL ≥130 mg/dL	Low	1.00	1.19 (0.55-2.57)	1.76 (0.83-3.71)	1.05 (0.48-2.33)
	High	1.15 (0.54-2.43)	2.13 (1.04-4.35)*	1.69 (0.82-3.50)	1.85 (0.90-3.81)**
HDL <40/50 mg/dL	Low	1.00	0.86 (0.50-1.49)	0.54 (0.31-0.92)*	0.59 (0.34-1.02)**
	High	1.37 (0.78-2.41)	0.96 (0.55-1.67)	1.00 (0.57-1.76)	0.80 (0.46-1.39)
Hypertension[c]	Low	1.00	1.31 (0.69-2.48)	1.21 (0.63-2.30)	1.72 (0.91-3.24)**
	High	1.45 (0.78-2.67)	1.85 (1.00-3.43)*	1.89 (1.02-3.49)*	1.69 (0.91-3.14)**
Dysglycemia	%MAs				
Diabetes	Low	1.00	2.11 (1.06-4.22)*	1.65 (0.82-3.30)	1.72 (0.86-3.44)
	High	0.43 (0.19-0.99)	1.28 (0.59-2.77)	0.79 (0.36-1.74)	0.67 (0.31-1.45)
Prediabetes	Low	1.00	1.37 (0.72-2.59)	0.61 (0.29-1.27)	1.06 (0.54-2.10)
	High	0.53 (0.26-1.10)	0.34 (0.13-0.84)*	0.99 (0.51-1.94)	**0.70 (0.34-1.42)**
Triglycerides ≥150 mg/dL	Low	1.00	1.62 (0.97-2.70)**	1.70 (1.00-2.89)*	**2.44 (1.43-4.15)***,#
	High	0.89 (0.52-1.55)	1.10 (0.62-1.95)	1.25 (0.73-2.14)	1.04 (0.60-1.79)
Cholesterol ≥200 mg/dL	Low	1.00	2.17 (1.16-4.03)*	2.29 (1.22-4.29)*	1.92 (1.01-3.67)*
	High	1.24 (0.63-2.42)	1.68 (0.85-3.32)	1.93 (1.02-3.67)*	1.76 (0.92-3.37)**
LDL ≥130 mg/dL	Low	1.00	2.33 (1.10-4.94)*	2.39 (1.12-5.11)*	2.44 (1.14-5.22)*
	High	1.93 (0.89-4.20)	2.02 (0.90-4.54)*	2.17 (0.99-4.72)**	1.45 (0.64-3.31)
HDL <40/50 mg/dL	Low	1.00	0.74 (0.42-1.29)	0.61 (0.35-107)**	0.62 (0.35-1.09)*
	High	0.72 (0.41-1.27)	0.60 (0.34-1.06)**	0.49 (0.28-0.85)*	0.42 (0.23-0.72)*
Hypertension	Low	1.00	1.67 (0.95-2.94)**	1.71 (0.96-3.06)**	1.49 (0.82-268)
	High	1.00 (0.54-1.85)	0.88 (0.46-1.69)	0.93 (0.51-1.70)	1.30 (0.71-2.37)

Cardiometabolic Outcome	As metabolism Indicator	Household water arsenic concentration quartile (µg/L)			
		**<25.5**	**≥25.5-<47.9**	**≥47.9-<79.0**	**≥79.0**
Dysglycemia	%iAs				
Diabetes	Low	1.00	2.03 (0.98-4.20)**	2.53 (1.62-6.08)**	2.03 (0.49-2.48)*
	High	1.09 (0.48-2.50)	3.32 (1.62-6.80)*	1.10 (0.49-2.48)	1.37 (0.64-2.92)
Prediabetes	Low	1.00	1.81 (0.86-3.85)	1.80 (0.86-2.91)	1.48 (0.69-3.19)
	High	2.14 (1.04-4.42)*	1.62 (0.73-3.58)	1.32 (0.59-2.94)	1.93 (0.91-4.07)**
Triglycerides ≥150 mg/dL	Low	1.00	1.53 (0.89-2.61)	1.41 (0.84-2.37)	**2.23 (1.31-3.79)** *
	High	0.74 (0.42-1.28)	0.99 (0.58-1.72)	1.19 (0.68-2.07)	**0.93 (0.54-1.62)** *,#
Cholesterol ≥200 mg/dL	Low	1.00	2.23 (1.20-4.15)*	2.17 (1.19-3.98)*	1.88 (1.02-3.49)*
	High	1.07 (0.55-2.09)	1.41 (0.73-2.70)	1.67 (0.87-3.22)	1.53 (0.80-2.91)
LDL ≥130 mg/dL	Low	1.00	1.69 (0.86-3.30)	1.60 (0.83-3.08)	1.38 (0.70-2.71)
	High	0.72 (0.33-1.54)	0.98 (0.48-2.03)	1.10 (0.52-2.30)	0.95 (0.46-1.99)
HDL <40/50 mg/dL	Low	1.00	0.80 (0.45-1.42)	0.71 (0.41-1.23)	0.67 (0.38-1.17)
	High	0.93 (0.52-1.63)	0.70 (0.40-1.23)	0.51 (0.29-0.90)	0.49 (0.28-0.85)
Hypertension	Low	1.00	1.43 (0.79-2.61)	1.40 (0.78-2.49)	1.30 (0.72-2.34)
	High	0.85 (0.46-1.57)	0.99 (0.53-1.82)	0.93 (0.50-1.74)	1.30 (0.70-2.38)
Dysglycemia	DMAs/MAs				
Diabetes	Low	1.00	2.96 (1.27-6.93)*	2.01 (0.83-4.84)	1.52 (0.64-3.59)
	High	2.49 (1.08-5.73)*	5.36 (2.38-12.08)*	3.85 (1.71-8.69)	4.33 (1.93-9.69)*
Prediabetes	Low	1.00	0.63 (0.25-1.59)	1.71 (0.82-3.56)	1.19 (0.56-2.53)
	High	1.58 (0.77-3.25)	2.31 (1.13-4.75)*	1.04 (0.47-2.29)	1.79 (0.85-3.80)
Triglycerides ≥150 mg/dL	Low	1.00	1.52 (0.85-2.72)	1.52 (0.86-2.66)	**1.31 (0.74-2.30)**
	High	1.37 (0.79-2.39)	1.90 (1.09-3.29)*	2.18 (1.24-3.84)*	**3.00 (1.71-5.27)** *,#
Cholesterol ≥200 mg/dL	Low	1.00	1.61 (0.82-3.19)	1.93 (1.00-3.72)*	1.69 (0.88-3.25)
	High	1.25 (0.64-2.45)	2.29 (1.20-4.35)*	2.35 (1.23-4.50)*	2.04 (1.06-3.92)*
LDL ≥130 mg/dL	Low	1.00	1.10 (0.56-2.54)	1.42 (0.68-2.94)	0.89 (0.41-1.94)
	High	0.93 (0.44-1.97)	1.74 (0.86-3.53)	1.67 (0.96-4.12)	1.75 (0.86-3.55)
HDL <40/50 mg/dL	Low	1.00	0.90 (0.51-1.59)	0.62 (0.36-1.07)**	0.57 (0.33-0.99)*
	High	1.34 (0.76-2.34)	0.91 (0.52-1.61)	0.89 (0.50-1.59)	0.83 (0.33-1.47)
Hypertension	Low	1.00	1.01 (0.52-1.96)	1.04 (0.55-1.97)	1.43 (0.77-2.68)
	High	1.16 (0.63-2.15)	1.78 (0.96-3.28)**	1.76 (0.94-3.49)**	1.58 (0.84-2.97)

Cardiometabolic Outcome	As metabolism Indicator	Household water arsenic concentration quartile (µg/L)			
		**<25.5**	**≥25.5-<47.9**	**≥47.9-<79.0**	**≥79.0**
Dysglycemia	MAs/iAs				
Diabetes	Low	1.00	3.18 (1.45-6.96)**	1.75 (0.79-3.87)	1.84 (0.84-4.05)
	High	0.71 (0.31-1.62)	1.34 (0.59-3.04)	1.26 (0.56-2.83)	1.05 (0.46-2.34)
Prediabetes	Low	1.00	1.12 (0.59-2.14)	0.65 (0.32-1.27)	0.79 (0.41-1.54)
	High	0.26 (0.12-0.55)*	0.31 (0.14-0.69)*	0.55 (0.28-1.09)*	0.49 (0.24-0.99)*
Triglycerides ≥150 mg/dL	Low	1.00	1.40 (0.81-2.41)	1.87 (1.08-3.23)*	1.74 (1.01-3.01)*
	High	1.26 (0.72-2.19)	1.91 (1.10-3.32)*	1.56 (0.91-2.69)	2.08 (1.20-3.62)*
Cholesterol ≥200 mg/dL	Low	1.00	1.79 (0.89-3.58)	2.70 (1.38-5.28)*	1.98 (0.99-3.95)**
	High	1.70 (0.86-3.39)	2.98 (1.52-5.84)*	2.34 (1.19-4.57)*	2.47 (1.25-4.86)*
LDL ≥130 mg/dL	Low	1.00	2.89 (1.21-6.93)*	3.70 (1.56-8.79)*	2.34 (0.95-5.79)**
	High	3.33 (1.42-7.83)*	3.54 (1.49-8.41)*	2.99 (1.26-7.11)*	3.37 (1.42-8.03)*
HDL <40/50 mg/dL	Low	1.00	0.86 (0.49-1.51)	0.53 (0.30-0.92)*	0.60 (0.35-1.05)**
	High	0.90 (0.51-1.57)	0.64 (0.36-1.13)	0.68 (0.39-1.18)	0.53 (0.30-0.92)*
Hypertension	Low	1.00	1.47 (0.80-2.71)	1.27 (0.69-2.32)	1.55 (0.84-2.85)
	High	1.08 (0.58-1.97)	1.22 (0.65-2.27)	1.36 (0.74-2.86)	1.36 (0.73-2.51)

*P<0.05 **P<0.10 for adjusted odds ratios (95% CI) for elevated cardiometabolic risk associated with increasing concentrations of water As combined with As metabolism indicators; the referent are individuals in the lowest quartile of water As with < median levels of each As metabolism indicator. Results come from multinomial or logistic models adjusted for age, gender, smoking status, alcohol consumer, BMI, elevated waist circumference and main water source (well, treatment plant, other). The referent group for all ORs is subjects with the metabolism indicator below the median in the lowest quartile of water As.

[#]P<0.10 indicates additive interaction between levels of each metabolism indicator and quartiles of water As (methods as in Vanderweele and Knoll 2014); values **bolded**.

[a]*Diabetes:* fasting plasma glucose (FPG) ≥126 mg/dL, 2h plasma glucose (2HPG) ≥200 mg/dL, or self-reported diabetes diagnosis or medication use. Pre-diabetes: FPG ≥110-<126 mg/dL or 2HPG ≥140 mg/dL. [b]*Median cutoffs* used to defined high vs. low concentrations of each As metabolism indicator as follows: %DMAs = 76.6%; %MAs = 14.1%; DMAs/MAs = 5.441; MAs/iAs = 1.579. [c]*Hypertension*: systolic blood pressure >140 mm Hg, diastolic blood pressure >90 or hypertensive medication use.
